# Subrenal capsule assay in selection of chemotherapy after operation for recurrent ovarian cancer.

**DOI:** 10.1038/bjc.1991.17

**Published:** 1991-01

**Authors:** P. Venesmaa, O. Ylikorkala

**Affiliations:** I and II Department of Obstetrics and Gynecology, Helsinki University Central Hospital, Finland.

## Abstract

Forty-six patients with recurrent ovarian cancer were reoperated, and cancer samples for the subrenal capsule assay (SRCA) were collected from 23 of them, whereas this test was not done in the remaining 23 control patients. The SRCA was evaluable in 22 cases (96%). Taken together, no significant difference appeared in the 3 years' survival figures between the groups: seven of 22 patients (32%) with the evaluable SRCA and six of 23 control patients (26%) were alive. However, a further analysis of the data revealed that the SRCA guided the selection of chemotherapy only in 15 patients, whereas tumour samples were resistant to all cytostatics tested in six cases and toxic side-effects limited the clinical application of the test results in the remaining one case. Four of the 11 patients (36%) whose further chemotherapy was strictly chosen based on the SRCA and seven of the 24 patients (29%) whose treatment was based on physician's choice survived at least 3 years. Our conclusion is that the SRCA is of limited value in the selection of second-line chemotherapy in recurrent ovarian cancer.


Br. J. Cancer (1991), 63, 84-86 © Macmillan Press Ltd., 1991

### Subrenal capsule assay in selection of chemotherapy after operation for recurrent ovarian cancer

P. Venesmaa & O. Ylikorkala

*I and II Department of Obstetrics and Gynecology, Helsinki University Central Hospital, Helsinki, Finland.*

**Summary** Forty-six patients with recurrent ovarian cancer were reoperated, and cancer samples for the subrenal capsule assay (SRCA) were collected from 23 of them, whereas this test was not done in the remaining 23 control patients. The SRCA was evaluable in 22 cases (96%). Taken together, no significant difference appeared in the 3 years' survival figures between the groups: seven of 22 patients (32%) with the evaluable SRCA and six of 23 control patients (26%) were alive. However, a further analysis of the data revealed that the SRCA guided the selection of chemotherapy only in 15 patients, whereas tumour samples were resistant to all cytostatics tested in six cases and toxic side-effects limited the clinical application of the test results in the remaining one case. Four of the 11 patients (36%) whose further chemotherapy was strictly chosen based on the SRCA and seven of the 24 patients (29%) whose treatment was based on physician's choice survived at least 3 years. Our conclusion is that the SRCA is of limited value in the selection of second-line chemotherapy in recurrent ovarian cancer.

The subrenal capsule assay (SRCA) (Bogden *et al.*, 1981) correctly predicts the response to cytostatic chemotherapy in 63-85% of patients with previously untreated ovarian cancer (Bogden, 1985; Griffin *et al.*, 1983; Mäenpää *et al.*, 1985*a,b*; Stratton *et al.*, 1984, 1986, 1988). The situation may differ in cases with recurrent cancer, when cell populations resistant to first-line chemotherapy have become selected out to form clinically detectable tumours (Mäenpää, 1985*c*). There are some data to suggest that the SRCA could be useful in the selection of the second-line chemotherapy (Griffin *et al.*, 1983; Mäenpää, 1985*c*), but so far no data exist on improved survival rates in patients whose second-line chemotherapy has been guided by the use of the SRCA. We therefore assessed the value of the SRCA in patients with recurrent ovarian cancer that were followed up to death or for a minimum of 3 years.

### Materials and methods

Forty-six patients between 25 and 76 years of age with recurrent ovarian cancer were studied (Table I). The primary operation was radical in seven women, whereas tumours smaller than 2 cm of size remained in nine women and tumours larger than 2 cm remained in 30 women. Serous cystadenocarcinoma was the most common type of cancer (22 women) followed by anaplastic cancer (11 women), miscellaneous cancer (nine women with mesonephric cancers and one with endometrioid cancer) and mucinous cystadenocarcinoma (three women) (Table I). The cancer had spread to clinical stages III-IV in 37 women. All patients following surgery had been treated with 4-28 courses of various cytostatics, mostly with the combination of doxorubicin, cisplatin and cyclophosphamide ($n = 34$ patients). Thirty-five had responded favourably to the therapy, as assessed by the standard criteria (Miller *et al.*, 1981), whereas in 11 women no response to therapy and/or progression of the disease was seen.

These 46 patients entered our study with the approval of the local committee of ethics between 5-48 months after primary operation, when they came for a routine second-look ($n = 32$) and third-look ($n = 6$) laparotomy or a debulking operation ($n = 8$). At operation 19 patients had residual tumour of size 2 cm or less (after operation no macroscopic tumour in six women), whereas 27 women had tumours larger than 2 cm (after operation no macroscopic tumour in four women and tumour less than 2 cm in six women). At this operation samples of histologically confirmed cancer were collected for the SRCA in 23 patients (operated on Monday-Wednesday), whereas this was not possible in 23 comparable control patients (operated on Thursday-Friday). The two groups were comparable as regards various clinical variables (Table I).

The tumour samples were put immediately into sterile tubes containing medium 199 and transported to the laboratory for the SRCA, as described in detail previously (Kangas & Perilä, 1985). The samples were cut into 1 $mm^3$ pieces and implanted under the renal capsules of 30 mice which were then treated with five various cytostatic combinations (five mice for each) or served as a control group (five mice). Cisplatin-doxorubicin-cyclophosphamide, cisplatin-hexamethylmelamine-melphalan and cisplatin-etoposide-hexamethylmelamine were the most frequent combinations tested (Table II). Response was judged by difference in tumour size between the untreated control and treated mice, and results were interpreted as sensitive if the mean tumour size had decreased by 1 omu (omu = ocular micrometer unit, 10 omu = 1 mm) or more, intermediately sensitive if the mean tumour size had decreased by less than 1 omu or increased by less than one-third of the $\Delta TS$ ($\Delta TS$ = difference between the initial and final tumour size) in the control group, and resistant if the mean $\Delta TS$ was one-third or more of that in the control group (Mäenpää *et al.*, 1985*b*).

The results of the SRCA were used in selecting chemotherapy after reoperation, whereas in the control group therapy was chosen based on clinical experience.

### Results

One patient had serous cystadenocarcinoma which did not grow in control mice and this test was discarded. Thus 22 of 23 tests (96%) were evaluable.

Cancer samples of ten patients (45%) were resistant to the combination of cisplatin-doxorubicin (or epiadriamycin)-cyclophosphamide or to the combination of cisplatin-hexamethylmelamine-melphalan (Table II). Taken together, four patients (18%) had sensitive tumours, 13 (59%) had tumours of intermediate sensitivity at least to one combination tested, whereas samples from six patients (27%) were resistant to all combinations tested (Table II).

The use of the SRCA directed the selection of chemotherapy totally in 11 patients and partly in four patients; in

Correspondence: P. Venesmaa, I and II Department of Obstetrics and Gynecology, Helsinki University Central Hospital, Haartmaninkatu 2, 00290 Helsinki, Finland.
Received 27 February 1990; and in revised form 16 August 1990.

**Table I** Clinical characteristics of the study population

| | *Primary operation* | *Reoperation SRCA done* | *Reoperation SRCA not done* |
|---|---|---|---|
| Number of patients | 46 | 23 | 23 |
| Age (median, range) | 55 (25–76) | 55 (25–76) | 55 (27–71) |
| Stage | | | |
| I–II | 9 | 6 | 3 |
| III–IV | 37 | 17 | 20 |
| Histology | | | |
| Serous cystadenocarcinoma | 22 | 12 | 10 |
| Mucinous cystadenocarcinoma | 3 | 2 | 1 |
| Anaplastic cancer | 11 | 5 | 6 |
| Miscellaneous cancer | 10 | 4 | 6 |
| Time from primary operation to reoperation (months) (median, range) | | 16 (7–47) | 16 (5–49) |
| Tumour size at reoperation | | | |
| Tumour size <2 cm | | 10 | 9 |
| Tumour size >2 cm | | 13 | 14 |
| Type of reoperation | | | |
| Second look | | 16 | 16 |
| Third look | | 3 | 3 |
| Debulking | | 4 | 4 |

**Table II** The sensitivity of the tumour in the SRCA in 23 patients in relation to previous and consequent cytotoxic treatment

| *Patient's initials* | *Drug combination before reoperation* | *Sensitivity of the tumour in the SRCA* | | | | | | | | | *Drug combination after reoperation* | *The SRCA directed chemotherapy* |
|---|---|---|---|---|---|---|---|---|---|---|---|---|
| | | *DDP A CTX* | *DDP HMM VP16* | *DDP HMM LPAM* | *DDP EPIA CTX* | *DDP A HMM* | *DDP EPIA HMM* | *DDP CTX ACLA* | *DDP 5-FU HMM* | *DDP MTX 5-FU* | | |
| M.A.* | DDP-A-CTX | I | I | I | – | – | – | – | – | – | DDP-A-CTX | Yes |
| M.G.* | DDP-A-CTX | I | I | I | – | – | – | – | – | – | DDP-A-CTX | Yes |
| P.A. | DDP-A-CTX | I | I | I | – | – | – | – | – | – | DDP-A-CTX | Yes |
| K.I. | DDP-A-CTX | I | I | S | – | I | – | I | – | – | DDP-A-CTX | Yes |
| M.M. | DDP-A-CTX | I | I | I | – | – | – | – | – | – | DDP-A-CTX | Yes |
| A.U.* | DDP-A-CTX | I | I | R | – | I | – | R | – | – | DDP-A-CTX-HMM | Yes |
| J.R.* | DDP-A-CTX | I | R | I | – | – | – | – | R | I | DDP-LPAM-HMM | Yes |
| T.A. | DDP-A-CTX | I | – | S | – | I | – | I | – | – | DDP-LPAM-HMM | Yes |
| R.A. | DDP-A-CTX | I | – | I | R | – | I | – | I | – | DDP-LPAM-HMM | Yes |
| A.T. | DDP-A-CTX | R | I | R | | R | – | R | – | – | DDP-VP16-HMM | Yes |
| T.N. | DDP-A-CTX | – | I | S | I | – | – | – | I | S | DDP-MTX-5-FU | Yes |
| B.A.* | DDP-CTX | S | I | I | – | I | – | I | – | – | DDP-CTX-HMM | Partly (see text) |
| F.A. | 5-FU-A-CTX | R | – | I | – | I | – | – | I | – | DDP-LPAM | Partly (see text) |
| P.M. | 5-FU-A-CTX | R | R | I | – | R | – | R | – | – | DDP-LPAM-VP16 | Partly (see text) |
| G.S. | DDP-A-CTX | I | I | R | – | – | – | – | – | – | CAR-HMM | Partly (see text) |
| I.A.* | DDP-VP16-HMM | R | R | R | – | – | – | – | – | – | DDP-VP16-HMM | No |
| H.K. | DDP-A-CTX | R | R | R | – | – | – | – | – | – | CAR-CTX | No |
| S.S. | DDP-A-CTX | R | R | R | – | – | – | – | – | – | DDP-CTX | No |
| U.I.* | DDP-A-CTX | R | R | R | – | – | – | – | – | – | DDP-A-CTX | No |
| R.S. | DDP-A-CTX | R | R | R | – | – | – | – | – | – | DDP-A-CTX | No |
| V.H. | DDP-A-CTX | R | R | R | – | – | – | – | – | – | DDP-A-CTX | No |
| Å.K. | DDP-A-CTX | – | R | R | I | – | – | – | R | R | MTX-5-FU | No |
| H.R.* | 5-FU-A-CTX | This test was discarded because tumour sample did not grow in control mice. | | | | | | | | | DDP-A-CTX | |

* = patient surviving 3 years; S = sensitive; I = intermediately sensitive; R = resistant to cytostatics in the SRCA; – = not tested; A = doxorubicin; Acla = aclarubicin; CAR = carboplatin; CTX = cyclophosphamide; DDP = cisplatin, EPIA = epiadriamycin; 5-FU = 5-fluorouracil; HMM = hexamethylmelamine; L-PAM = melphalan; MTX = methotrexate; VP-16 = etoposide.

the latter patients some drugs were excluded because they caused intolerable side-effects in these patients (Table II). Thus 15 patients received chemotherapy guided by the SRCA, whereas in seven patients the treatment was continued based on the physician's choice.

After relaparotomy, on average seven courses (range 1–20) of chemotherapy were given to the SRCA group and the control group also had seven courses (range 1–16). The 'routine' cisplatin-doxorubicin-cyclophosphamide was used more often in patients whose treatment was not based on the SRCA ($n = 17$) than in those who underwent the SRCA ($n = 9$) (Tables II and III). Nine of the 22 patients with the SRCA (41%) and 15 of the 23 control patients (65%) were treated with the same cytostatic combinations used before the study operation. Six patients with cancer resistant to all combinations tested were treated with similar combination to those employed after primary operation.

Fifteen patients have died in the SRCA group and 17 in the control group, and this occurred on average 11 months after reoperation in both groups. Four of 11 patients with SRCA guided chemotherapy (36%) and seven of 24 patients with physician's choice of chemotherapy (29%) (patient H.R. with unevaluable SRCA was also included in this group) survived at least 3 years (Table IV). After 3 years three patients with SRCA guided chemotherapy (27%) and six patients (25%) with physician's choice of chemotherapy were free of disease.

### Discussion

There is a great interest in tests which can be of help in selection of cytotoxic treatment for recurrent ovarian cancer. One of them was the human tumour stem cell assay (Ham-

**Table III** Cytotoxic treatment of 23 control patients before and after reoperation

| *Number of patients* | *Drug combination before operation* | *Drug combination after reoperation* |
|---|---|---|
| 14 (6 survived) | DDP-A-CTX | DDP-A-CTX |
| 1 | DDP-A-CTX | DDP-CTX |
| 1 | DDP-A-CTX | DDP-VLB |
| 1 | DDP-A-CTX | MTX-A-5-FU |
| 2 | 5-FU-A-CTX | DDP-A-CTX |
| 1 | 5-FU-A-VCR-CTX | DDP-A-CTX |
| 1 | VCR-A-CTX | VCR-A-5-FU |
| 1 | VCR-CTX | VCR-CTX |
| 1 | VCR-CTX | DDP-BLEO-VLB |

BLEO = bleomycin; VLB = vinblastine; VCR = vincristine, other abbreviations: see Table II.

**Table IV** The rates of survival at 3 years' follow-up in patients who were treated with and without the SRCA test

| | *SRCA guided chemotherapy* | *Physician's choice of chemotherapy* |
|---|---|---|
| Total patients treated | 4/11 (36%) | 7/24** (29%) |
| DDP-A-CTX after reoperation* | 3/6 (50%) | 7/17 (45%) |
| Other therapy | 1/5 (20%) | 0/6 (0%) |

DDP-A-CTX = cisplatin-doxorubicine-cyclophosphamide. *One patient with cisplatin-doxorubicin-cyclophosphamide-hexamethylmelamine included. **Patient with unevaluable SRCA test included.

burger & Salmon, 1977) which predicted correctly drug resistance in 90–99% of cases (Alberts *et al.*, 1980; Welander *et al.*, 1983) but failed to detect drug sensitivity in approximately 40% of cases (Alberts *et al.*, 1980). The clinical usefulness of this test is limited by inadequate tumour cell growth in culture (Selby *et al.*, 1983) and the long incubation period (up to 2–3 weeks) required in the test. The SRCA is readable in 6 days and it is applicable to test tumour sensitivity to several cytostatics or their combinations (Stratton *et al.*, 1984; Mäenpää *et al.*, 1985*b*). Furthermore, this test can be used to assess the potential effect of cytostatics which require bioactivation (Stratton *et al.*, 1984).

In spite of the fact that the SRCA was evaluable in the majority of patients, it could help in guiding the chemotherapy only in half of the patients and a considerable portion of patients in both study groups actually received the same combinations to those used after primary operation. This was partly due to the fact that some drugs which appeared effective according to the SRCA could not be used in patients, because they had caused intolerable side-effects. There are also several other factors which may explain the lack of any profound effect of the use of the SRCA on the survival figures. First, ovarian cancer is a heterogenous tumour consisting of a number of different cell types, and it is impossible to guarantee that a small tumour piece taken blindly at operation was the most representative part of the cancer. Second, the majority of our patients received as first-line therapy the combination of cisplatin-doxorubicin-cyclophosphamide which is one of the most potent drug combinations used in the treatment of ovarian cancer; this is also clearly seen from our data. Thus, whatever new chemotherapy alternatives were suggested by the use of the SRCA, they are likely to prove less effective than the combination of cisplatin-doxorubicin-cyclophosphamide. And third, cancer cells are known to develop resistance to multiple drugs simultaneously, perhaps as a result of cell membrane glycoprotein which promotes the efflux of drugs from cancer cells (Scheper *et al.*, 1988). In the future when more effective and possibly more selective antitumour drugs are available, the SRCA may potentially have a role in the management of advanced ovarian cancer.

This study was supported partly by the Emil Aaltonen Foundation and The Finnish Social Insurance Institution. The subrenal capsule assays were performed in the laboratory of Farmos Group Research Center in Turku, Finland.

### References

ALBERTS, D.S., SALMON, S.E., CHEN, H.S.G. & 3 others (1980). *In vitro* clonogenic assay for predicting response of ovarian cancer to chemotherapy. *Lancet*, **11** 340.

BOGDEN, A.E., COBB, W.R., LEPAGE, D.J. & 5 others (1981). Chemotherapy responsiveness of human tumours as first transplant generation xenografts in the normal mouse. *Cancer*, **48**, 10.

BOGDEN, A.E. (1985). The Subrenal Capsule Assay (SRCA) and its predictive value in oncology. *Ann. Chir. Gynaecol.*, **74**, Suppl 199, 12.

GRIFFIN, T.W., BOGDEN, A.E., REICH, S.D. & 6 others (1983). Initial clinical trials of subrenal capsule assay as a predictor of tumour response to chemotherapy. *Cancer*, **52**, 2185.

HAMBURGER, A.W. & SALMON, S.E. (1977). Primary bioassay of human tumour stem cells. *Science*, **97**, 461.

KANGAS, L. & PERILÄ, M. (1985). Clinical praxis and laboratory procedures in Subrenal Capsule Assay (SRCA). *Ann. Chir. Gynaecol.*, **74**, Suppl. 199, 7.

MILLER, A.B., HOOGSTRATEN, B., STAQUET, M. & WINKLER, A. (1981). Reporting results of cancer treatment. *Cancer*, **47**, 207.

MÄENPÄÄ, J., GRÖNROOS, M. & KANGAS, L. (1985*a*). Subrenal Capsule Assay in choosing cytostatics for gynaecological tumours. *Ann. Chir. Gynaecol.*, **74**, Suppl 199, 28.

MÄENPÄÄ, J., KANGAS, L. & GRÖNROOS, M. (1985*b*). Response of ovarian cancer to combined cytotoxic agents in the Subrenal Capsule Assay: Part I. *Obstet. Gynecol.*, **66**, 708.

MÄENPÄÄ, J. (1985*c*). Subrenal Capsule Assay as predictor of clinical response of ovarian cancer to chemotherapy. Part II. *Obstet Gynecol.*, **66**, 714.

SCHEPER, R.J., BULTE, J.W.M., BRAKKEE, J.G.P. & 8 others (1988). Monoclonal antibody JSB-1 detects a highly conserved epitope on the P-glycoprotein associated with multi-drug-resistance. *Int. J. Cancer*, **42**, 389.

SELBY, P., BUICK, R.N. & TANNOCK, I. (1983). A critical appraisal of the 'human tumour stem-cell assay'. *N. Engl. J. Med.*, **308**, 129.

STRATTON, J.A., KUCERA, P.R., MICHA, J.P. & 4 others (1984). The Subrenal Capsule Tumor implant as a predictor of clinical response to chemotherapy: three years of experience. *Gynecol. Oncol.*, **19**, 336.

STRATTON, J.A., KUCERA, P.R., RETTENMAIER, M.A. & 5 others (1986). Accurate laboratory predictions of patients with advanced ovarian cancer to treatment with cyclophosphamide, doxorubicin, and cisplatin. *Gynecol. Oncol.*, **25**, 302.

STRATTON, J.A., RETTENMAIER, M.A., KUCERA, P.R., BERMAN, M.L. & DISAIA, P.J. (1988). Concordance of combination and single agent chemosensitivity prediction in ovarian carcinoma using the subrenal capsule xenograft assay (SRCA). *Gynecol. Oncol.*, **30**, 416.

WELANDER, C.E., HOMESLEY, H.D. & JOBSON, V.W. (1983). *In vitro* chemotherapy testing of gynecologic tumours: basis for planning therapy? *Am. J. Obstet Gynecol.*, **147**, 188.


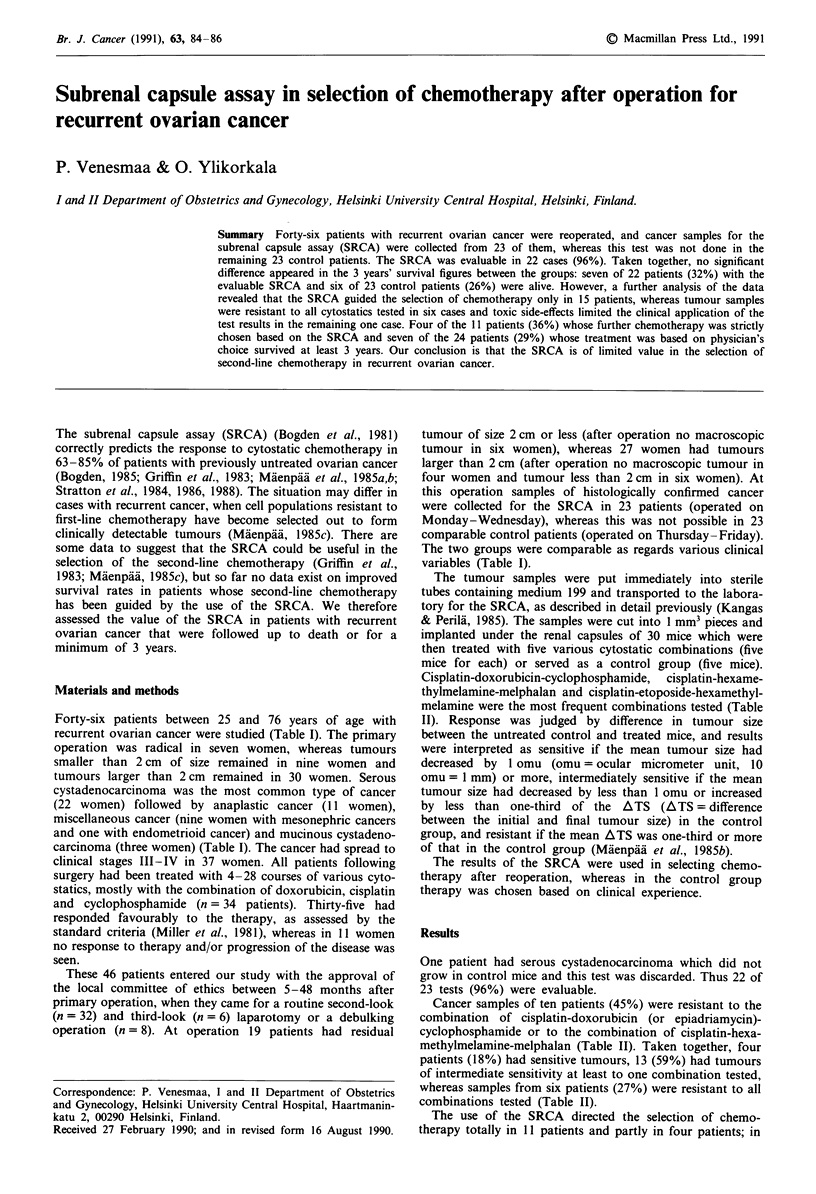

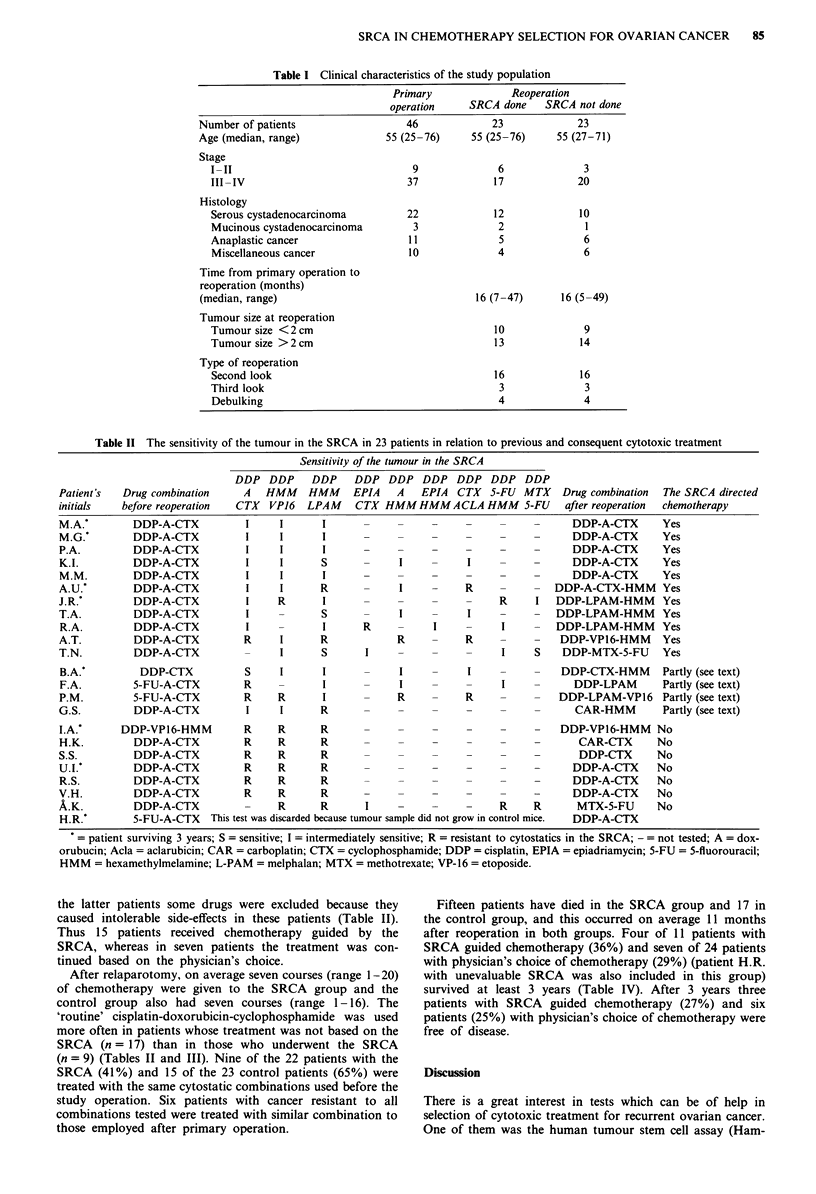

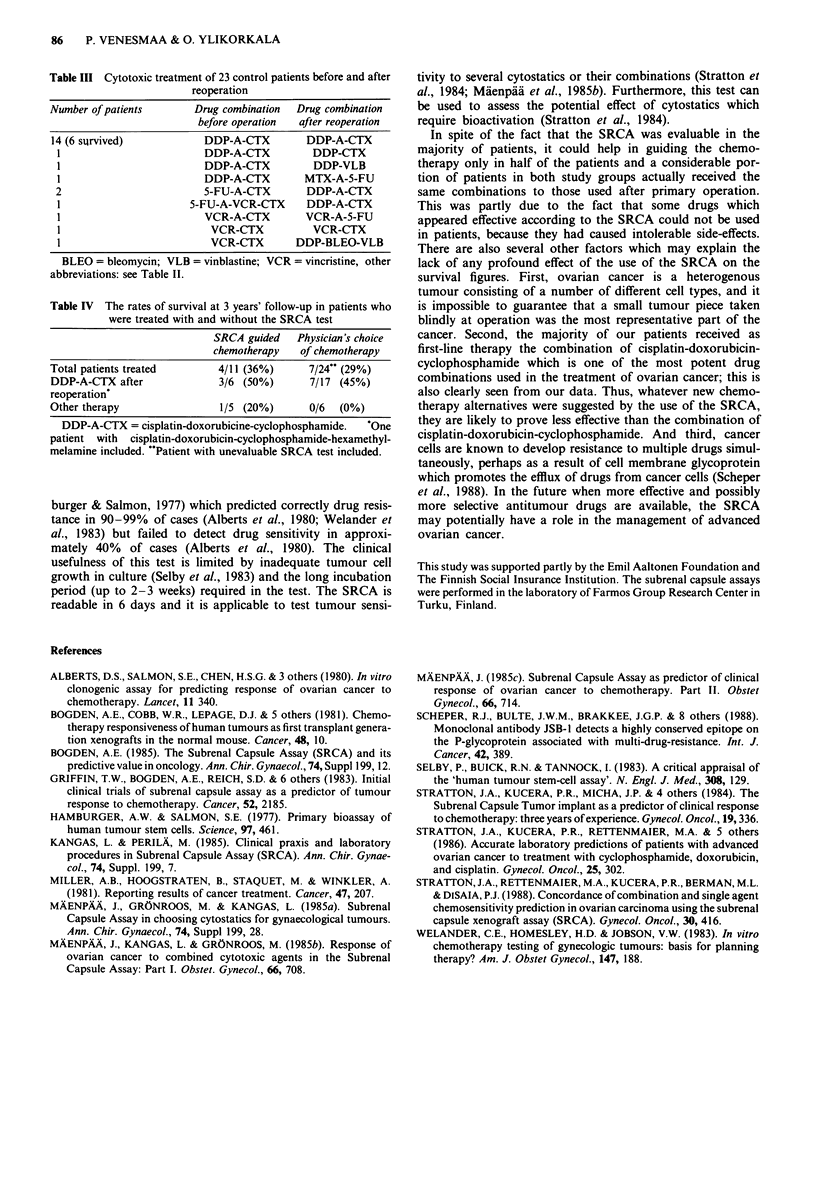


## References

[OCR_00404] Griffin T. W., Bogden A. E., Reich S. D., Antonelli D., Hunter R. E., Ward A., Yu D. T., Greene H. L., Costanza M. E. (1983). Initial clinical trials of the subrenal capsule assay as a predictor of tumor response to chemotherapy.. Cancer.

[OCR_00409] Hamburger A. W., Salmon S. E. (1977). Primary bioassay of human tumor stem cells.. Science.

[OCR_00418] Miller A. B., Hoogstraten B., Staquet M., Winkler A. (1981). Reporting results of cancer treatment.. Cancer.

[OCR_00427] Mäenpä J., Kangas L., Grönroos M. (1985). Response of ovarian cancer to combined cytotoxic agents in the subrenal capsule assay: Part I.. Obstet Gynecol.

[OCR_00432] Mäenpä J. (1985). Subrenal capsule assay as a predictor of clinical response of ovarian cancer to chemotherapy: Part II.. Obstet Gynecol.

[OCR_00437] Scheper R. J., Bulte J. W., Brakkee J. G., Quak J. J., van der Schoot E., Balm A. J., Meijer C. J., Broxterman H. J., Kuiper C. M., Lankelma J. (1988). Monoclonal antibody JSB-1 detects a highly conserved epitope on the P-glycoprotein associated with multi-drug-resistance.. Int J Cancer.

[OCR_00443] Selby P., Buick R. N., Tannock I. (1983). A critical appraisal of the "human tumor stem-cell assay".. N Engl J Med.

[OCR_00447] Stratton J. A., Kucera P. R., Micha J. P., Rettenmaier M. A., Braly P. S., Berman M. L., DiSaia P. J. (1984). The subrenal capsule tumor implant assay as a predictor of clinical response to chemotherapy: 3 years of experience.. Gynecol Oncol.

[OCR_00451] Stratton J. A., Kucera P. R., Rettenmaier M. A., Dobashi K., Micha J. P., Braly P. S., Berman M. L., DiSaia P. J. (1986). Accurate laboratory predictions of the clinical response of patients with advanced ovarian cancer to treatment with cyclophosphamide, doxorubicin, and cisplatin.. Gynecol Oncol.

[OCR_00457] Stratton J. A., Rettenmaier M. A., Kucera P. R., Berman M. L., DiSaia P. J. (1988). Concordance of combination and single agent chemosensitivity prediction in ovarian carcinoma using the subrenal capsule xenograft assay (SRCA).. Gynecol Oncol.

[OCR_00463] Welander C. E., Homesley H. D., Jobson V. W. (1983). In vitro chemotherapy testing of gynecologic tumors: basis for planning therapy?. Am J Obstet Gynecol.

